# Two Variants of the *ANK1* Gene Associated with Hereditary Spherocytosis

**DOI:** 10.3390/biomedicines13020308

**Published:** 2025-01-27

**Authors:** Dżamila M. Bogusławska, Justyna Rybka, Paulina Koszela, Kazimierz Kuliczkowski, Aleksander F. Sikorski

**Affiliations:** 1 Department of Biotechnology, Institute of Biological Sciences, University of Zielona Góra, 65-516 Zielona Góra, Poland; d.boguslawska@wnb.uz.zgora.pl (D.M.B.); p.koszela@o2.pl (P.K.); 2 Department and Clinic of Hematology, Cellular Therapies and Internal Medicine, Wroclaw Medical University, 50-367 Wroclaw, Poland; justyna.rybka@umw.edu.pl; 3 Silesian Park of Medical Technology Kardio-Med Silesia, 41-800 Zabrze, Poland; kazkul@wp.pl; 4 Research and Development Centre, Regional Specialist Hospital, 51-154 Wroclaw, Poland

**Keywords:** hereditary spherocytosis, ankyrin-1, erythrocyte membrane protein, whole exome sequencing

## Abstract

**Background** Hereditary spherocytosis (HS) is an erythrocytic membranopathy that belongs to a group of rare genetic disorders. Mutations in five genes, including *ANK1*, cause clinical manifestations of the disease. Identified variations in individual families provide a better understanding of the molecular basis of the disease. **Methods** In this study, we used two sequencing methods, whole exome sequencing (WES) and Sanger sequencing, analyzing gDNA and cDNA as templates, to detect and verify the variants putatively responsible for the clinical symptoms observed in a Polish family diagnosed with HS. **Results** We detected two variants that occur in *cis* in the *ANK1* gene, a known missense mutation (NP_000028.3:p.V463I) and a novel frameshift mutation (NP_000028.3: p.V1626fs*64) that appears to be crucial for the probands. As shown by transcriptome studies, the mutant allele is not present at a detectable level. **Conclusions** We conclude that the molecular basis of this case is related to an unstable transcript of the mutant allele and that the direct cause of the HS is a deficiency of erythrocyte ankyrin leading to a disruption of the AE1-erythrocyte ankyrin-spectrin complex in the erythrocyte membrane.

## 1. Introduction

The most common erythrocyte membrane disorder in the world is hereditary spherocytosis, a hemolytic anemia that affects one in 2000–5000 people in northern European countries [1,2]. Disruption of the connections between the membrane skeleton and the lipid bilayer containing integral proteins is the molecular basis of disease [3,4]. In HS cases described in the literature, dysregulation or loss of erythrocyte membrane proteins can reduce the stability and deformability of erythrocytes [1]. The result is increased osmotic fragility of erythrocytes, leading to erythrocyte rupture, premature hemolysis, and anemia [5]. The lifespan of red blood cells (RBC) is reduced from 120 to an average of a dozen days [6].

Compensation for hemolysis by increased erythropoiesis usually depends on the type of defect, general health, and individual patients’ abilities. However, in most cases, it is insufficient, ultimately leading to anemia. Other diagnostic criteria include jaundice, reticulocytosis, splenomegaly, gallstones, spherocytes in the peripheral blood smear, and decreased osmotic resistance of erythrocytes [7,8]. The uniqueness of the variants underlying HS (often identified only within a single family), the presence of additional genetic variants, and similar clinical manifestations of other RBC defects as well as the general health of patients make the diagnosis of HS a challenge for hematologists [9,10]. 

Interesting research by Huisjes et al. shows that in patients from one family in which the defect correlates with moderate/severe spherocytosis, a greater diversity of clinical symptoms is observed [5]. In these patients, the lower density (correlated with lower MCHC) and heterogeneity of the erythrocytes caused relatively little membrane loss. Nevertheless, these cells were rapidly removed by the spleen due to a significant loss of membrane deformation capacity. In HS patients after splenectomy and with a mild form of HS, erythrocytes that remained in circulation for a longer time were characterized by higher density associated with significant membrane loss. The greatest diversity in severity and clinical manifestations of the HS was seen in patients with *ANK1* gene mutations. 

It is currently recognized that mutations in five proteins responsible for these interactions correlate with the RBC defect discussed here. These are proteins encoded by the following genes: *ANK1* (ankyrin-1), *SPTA1* (α-spectrin), *SPTB* (β-spectrin), *SLC4A1* (solute carrier family 4 member 1, Diego blood group, AE1), and *EPB42* (protein 4.2) [11,12]. Autosomal dominant inheritance is common in HS, and specific variants have been identified in the genes *ANK1* (MIM#182900), *SPTB* (MIM#616649), and *SLC4A1* (MIM#612653) in about two-thirds of cases [2,13]. However, biallelic pathogenic variants (a recessive form of HS) have been observed mainly in the *SPTA1* gene (MIM#270970) and less frequently in the *EPB42* (MIM#612690) and *ANK1* genes. α-Spectrin is a protein with high expression in human and mouse erythrocytes, which results in normal protein levels in heterozygotes. It is regulated by regulatory elements (GATA-1 and NF-E2) that determine the high erythroid-specific expression [14]. Heterozygotes of the *EPB42* gene are also clinically normal, whereas homozygotes have reduced levels of the AE1 protein, which is essential for erythrocyte membrane stability [15,16].

Among *de novo* mutations, changes in the *ANK1* gene predominate in patients with HS [17,18,19]. The gene encoding ankyrin-1 appears to be particularly prone to slipped strand mispairing during DNA replication [20]. This is due to the high GC content in the nucleotide sequence encoding the exons, especially in the *ANK1* gene promoter (77% between positions −1 to −306). The GC-rich ankyrin-1 gene promotor, like housekeeping promoters, does not contain consensus TATA, InR, or CCAAT sequences [21,22].

The *ANK1* gene encodes ankyrin-1, which links the spectrin-actin cytoskeleton to integral membrane proteins of the RBC and is essential for cell membrane integrity. The protein was first detected in erythrocytes, hence the name erythrocyte ankyrin is often used [23]. Further discoveries have shown their presence also in the muscle and brain [24,25]. Mutations in the *ANK1* gene can affect the expression of ankyrin-1 and disrupt the function of key membrane proteins. The absence or decrease of normal ankyrin-1 in the cell membrane also leads to a loss of β-spectrin [26]. Ankyrin mRNA deficiency resulting from mutations in the *ANK1* gene leads to reduced ankyrin synthesis, which in turn leads to reduced spectrin assembly at the membrane. The deficiency of these two crucial proteins affects the structure and flexibility of the RBC membrane, causing changes in the shape of the cell. 

Here we report a novel p.V1626fs*64 frameshift mutation and a known missense p.V463I mutation in the *ANK1* gene, detected by WES and confirmed by standard Sanger sequencing, related to the phenotype in a Polish family with autosomal dominant HS. Transcript analysis of this gene revealed only an unmutated sequence. The most probable cause of the molecular basis is the unstable transcript of the mutant allele and decreased ankyrin-1 in the erythrocytes of two HS patients from the studied AM family.

## 2. Materials and Methods

### 2.1. Patients 

The present study included two patients from a Polish family referred to the Department and Clinic of Hematology, Cellular Therapies, and Internal Medicine at the Medical University of Wroclaw, clinically diagnosed with HS. Moderate disease symptoms were seen in the un-splenectomized subjects: father (AM175, age 82) and daughter (AM174, age 52). Other family members, except for AM173 (daughter of the patient AM174, 16 years old, who was in the study and in a clinically healthy state), were not available. Typical features such as anemia, spherocytes on peripheral blood smear, splenomegaly, elevated bilirubin, and reticulocytosis were used as diagnostic criteria for HS. Clinical manifestations were similar between patients. This research received the approval of the Wroclaw Medical University Ethics Committee (protocol KB-199/2017). Before inclusion in the protocol, informed consent was obtained from all family members.

### 2.2. DNA and RNA Isolation 

Whole blood was obtained by venipuncture from two HS patients (AM175 and AM174) and one asymptomatic family member (AM173) and collected in tubes containing EDTA. Genomic DNA extraction was carried out using the standard method (QIAamp DNA Blood Mini Kit, Qiagen, Hilden, Germany). The miRNeasy Mini Kit (Qiagen, Hilden, Germany) was used to purify total RNA from fresh whole blood. The manufacturer’s recommendations have been followed for the above procedures. The isolated gDNA was stored at −20 °C and the RNA at −80 °C until analysis. Absorbance in a Cary 60 UV spectrophotometer at 260 nm was used to determine the concentration and purity of DNA and RNA samples.

### 2.3. Whole Exome Sequencing 

WES of the genomic DNA samples from three family members was performed at the Heflin Center for Genomic Science Core Laboratories at the University of Alabama at Birmingham (Birmingham, AL, USA). Briefly, the sequencing library was prepared using the SureSelect Target Enrichment System (Agilent Technologies, Inc., Santa Clara, CA, USA) and captured using the Agilent SureSelect Human Clinical Research Exome (CRE) capture kit (Agilent Technologies, Inc., Santa Clara, CA, USA). The read length of each sample was 100 bp on an Illumina NextSeq500 (Illumina, Inc., San Diego, CA, USA), and the average coverage depth was at least 100×. 

### 2.4. Whole-Exome Sequencing Data Analysis

The WES data were trimmed for low-quality sequences and mapped against the UCSC Human Reference Genome (human genome 19/GRCh37.13) using Burrows-Wheeler Alignment (BWA). The Heflin Center for Genomic Science Core Laboratories at the University of Alabama at Birmingham (UAB), Birmingham, AL, USA performed bioinformatics analysis to detect single nucleotide variants (SNVs) and insertions/deletions. All variants were then filtered, annotated, and evaluated using Ingenuity Variant Analysis (IVA; QIAGEN, Redwood City, CA, USA).

A strategy was developed to select the rare and potentially deleterious variants present in both HS patients and lacking in a healthy family member. We mainly considered heterozygous and homozygous in-exon variations with predicted consequences (splicing, non-synonymous, frameshift, stop). The clinical significance of the selected changes was assessed using a 1000 Genome Browser database (https://www.internationalgenome.org), ClinVar (http://www.ncbi.nlm.nih.gov/clinvar/), HGMD (http://www.hgmd.cf.ac.uk/), Online Mendelian Inheritance in Man (OMIM) (http://www.omim.org/), Single Nucleotide Polymorphism (dbSNP) (https://www.ncbi.nlm.nih.gov/snp/), GeneCards (https://www.genecards.org/), The Universal Protein Resource (UniProt) (https://www.uniprot.org/) databases (each accessed on 27 November 2024), and literature data. 

### 2.5. Variants Validation 

Targeted Sanger sequencing of genomic DNA and/or cDNA was applied to validate potentially pathogenic relevant variants selected by WES. Total RNA was reversely transcribed into cDNA for sequencing using the Maxima H Minus Double-Stranded cDNA Synthesis Kit (Thermo Fisher Scientific, Waltham, MA, USA). Genomed S.A. (Warsaw, Poland) performed the Sanger sequencing and synthesized all primers. Primers used were designed with Gene Runner version 6.5.52x64 Beta. The sequences of all primers are listed in Tables S1.1 and S1.2 (see Supplementary Materials for details). PCR reaction parameters are listed in Tables S1.4 and S1.5. All the genetic variants reported in the manuscript have been lifted to GRCh38. 

## 3. Results

### 3.1. Hematological Parameters

Clinical manifestations, family history, laboratory test results, and morphological review of the peripheral blood smear were made to diagnose HS in the investigated family. The two studied probands (AM174 and AM175), who have not undergone splenectomy, presented similar clinical presentation resembling those of HS patients, namely, jaundice, moderate chronic anemia, reticulocytosis, bilirubinemia, and splenomegaly. The hematological parameters for probands are listed in Table S1.6. 

### 3.2. Validation of WES Results

For each available AM family member, WES was performed: including two symptomatic HS patients, AM174, the father of AM175, and one asymptomatic family member — the daughter of AM174, named here AM173. All the detected variants, which were filtered and annotated by Ingenuity Variant Analysis software, were classified according to their clinical significance using the ClinVar database and/or the ACMG classification [27]. Briefly, 93,162 nucleotide sequence changes were detected in the genetic material of AM family members using the WES method. We began the search of the obtained data by reviewing the pool of known and well-characterized genetic variants. The 1916 variants classified by the Human Gene Mutation Database (HGMD) were identified, including 1052 that were present simultaneously in both studied HS patients (AM174 and AM175). The only variant classified as pathogenic and detected as heterozygous in both HS patients was a frameshift mutation in the *ENAM* gene (p.V1626fs*64; HGMD CI033730 (DM)), which is responsible for autosomal recessive amelogenesis imperfecta and localized enamel defects. This change cannot be the basis of the analyzed HS case due to the inheritance pattern. None of above-mentioned known variants (classified by HGMD) appear to be crucial for the HS phenotype observed in the AM family.

**Figure 1:**
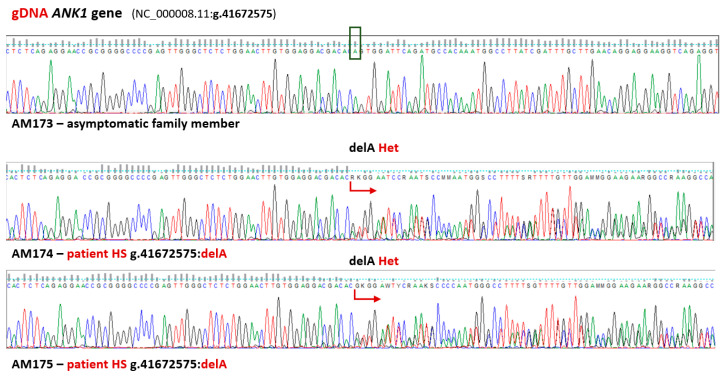
**The novel frameshift mutation of the *ANK1* gene is related to hereditary spherocytosis.** Sequence fragments of the *ANK1* gene in affected patients (AM174 and AM175) as well as one asymptomatic family member (AM173). Genomic DNA sequence analysis revealed a single nucleotide deletion only in HS patients (NM_000037.4:c.4959del causing the frameshift mutation NP_000028.3: p.V1626fs*64 resulting in a premature stop codon). The sequence compatible with the reference sequence is indicated by the green color of the boxes. The red arrows indicate the position of the pathogenic variant in the sequence.

Secondly, to investigate the correlation between the phenotype and genetic variations, we analyzed genes associated with the phenotype of known hereditary hemolytic anemias [28,29,30]. Data obtained by the WES method were compared with data collected from biomedical databases and the world literature regarding erythrocyte defects: membranopathies, enzymopathies, and hemoglobinopathies. In the panel of genes recommended for RBC pathologies, more than three hundred and sixty variants (including 11 unknown) have been identified (see Additional file S2 for details) [28]. Among these variants, we selected 12 potentially important changes in eight genes (correlated with membranopaties and enzymopathies as well as other potentially pathogenic variants) and verified their presence in the gDNA (Table S1.3). Selection criteria were inheritance pattern, frequency, clinical manifestation, and significance as classified by ClinVar, CADD score, and SIFT function prediction, as well as other data deposited in biomedical databases including HGMD and literature data. We applied IVA-recommended filtering algorithms (Common Variants, Predicted Deleterious, Phenotype Driven Ranking, and Biological Context). Among the detected variants, only one was crucial and classified as likely pathogenic according to ACMG recommendations. A novel *ANK1* gene c. 4959del (p.V1626fs*64) frameshift mutation was detected only in HS patients from the AM family (Figure 1).

Five genes associated with clinical phenotypes of HS were of particular interest for our research (*ANK1*, *SPTA1*, *SPTB*, *SLC4A1*, and *EPB42*). In four of these genes, 92 variants were found (see Additional file S3 for details). From these, we selected five potentially crucial changes (including the new variant (p.V1626fs*64), described above) in two genes (*ANK1* and *SPTB*), whose presence we verified in the gDNA (Additional file S1: Table S1.1). Two variants of the *SPTB* gene were only confirmed in a single AM family member: AM175 (heterozygous, NP_001342365.1:p.His1691Tyr, rs2082307149; frequency: A = 0.000007 (1/140244, GnomAD)) and AM173 (homozygous, asymptomatic)/AM 174 (heterozygous, HS patient) (NP_001342365.1:p.Arg1403Gln, rs17180350, CM187439 (DM?)). 

Two variants in the *ANK1* gene were confirmed only in HS patients AM174 and AM175. First, which is a known missense mutation (NM_000037.4:c.1387G>A, NP_000028.3:p.V463I, rs140085544) and was heterozygous in both HS patients. The second was a new single nucleotide deletion (NM_000037.4:c. 4959del, NP_000028.3: p.V1626fs*64), which is also heterozygous, in both HS patients, resulting in a frameshift and a premature stop codon sixty four codons downstream this change (see Figure 1). The above-mentioned missense mutation was first described by Eber et al. [31] and deposited in the HGMD database (CM960064) and is now classified in ClinVar (variant-condition record (RCV)) as “conflicting pathogenicity classifications” (uncertain significance(2); benign(1); probably benign(1); variant ID: 719959) [27]. The new variant (p.V1626fs*64) detected by WES was automatically classified as likely pathogenic using the results of the Ingenuity Variant Analysis plugin results based on the guidelines of the American College of Medical Genetics and Genomics and the Association for Molecular Pathology [25]. Criteria for classification of the p.V1626fs*64 variant were the following: PVS1, null variant (frameshift) in a gene where LOF is a known mechanism of disease (Very strong); PM2, absent from controls in the Exome Sequencing Project, 1000 Genomes Project, or Exome Aggregation Consortium (Moderate); PM4, protein length changes as a result of in-frame deletions in a nonrepeat region (Moderate); PP1, co-segregation with disease (HS) in multiple affected family members in a gene definitively known to cause the disease (Supporting); and PP4, the patient’s phenotype or family history is highly specific for a disease with a single genetic etiology (Supporting). The IVA plugin (QIAGEN, Redwood City, CA, USA) was used to predict the conservation phyloP *p*-value of 5.998E-10 for the variant (see Supplementary File S3 for details). 

**Figure 2:**
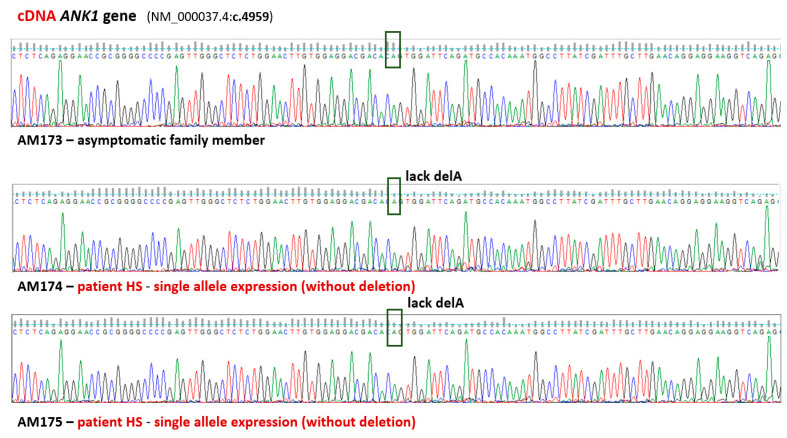
**Mutated transcripts are absent in patients’ transcriptomes.** Fragment of sequencing traces detected in AM family members showing loss of the mutant allele (NM_000037.4:c.4959del) in the cDNA compared to the genomic DNA. The sequence compatible with the reference sequence is indicated by the green color of the boxes.

The detected deletion (NM_000037.4:c.4959del, NP_000028.3: p.V1626fs*64) is in the sequence encoding the regulatory domain of erythrocyte ankyrin (1383’1881 amino acid residues). A transcript of this gene was examined to verify allelic expression. Analysis of the double-stranded cDNA based on Sanger sequencing revealed the expression of only one unmutated allele of the *ANK1* gene in both HS patients. Therefore, both mutations detected in the *ANK1* gene were verified: a frameshift mutation (p.V1626fs*64) and a missense mutation (p.V463I) (see Figure 2 and Figure S1.1). 

## 4. Discussion

Our previous research into the molecular basis of hereditary hemolytic anemias has allowed us to detect new variants and describe the molecular basis of these rare diseases in the Polish population [32,33,34,35,36,37]. Here, we report a Polish family with two variants in the *ANK1* gene: one is a known missense mutation and the other a newly discovered frameshift mutation.

We decided to use WES, which is currently the most effective method for identifying the molecular mechanism of inherited disorders that may underline the clinical symptoms seen in the probands from the AM family studied [29,30,38]. We were able to filter the variants identified by WES and narrow the search to changes associated with red cell pathology using the Ingenuity Variant Analysis plugin. The analysis allowed the selection of eight variants correlated with the phenotype of hereditary hemolytic anemias and four other potentially pathogenic changes that may have affected the health status of AM family members (see Table S1.2). These variations were verified in the gDNA by Sanger sequencing. Five changes in membranopathy-related genes were confirmed, including two each in the *ANK1* and *SPTB* genes. These four changes are in genes encoding proteins whose defects underlie HS, which is consistent with the initial clinical diagnosis. After analysis of all the data collected on these membranopathy-associated variants, two located in the *ANK1* gene were selected as primary candidates in the HS patients from the AM family. The four other potentially pathogenic variations listed in Table S1.2 appeared irrelevant to the hematological abnormalities observed in the probands.

**Figure 3:**
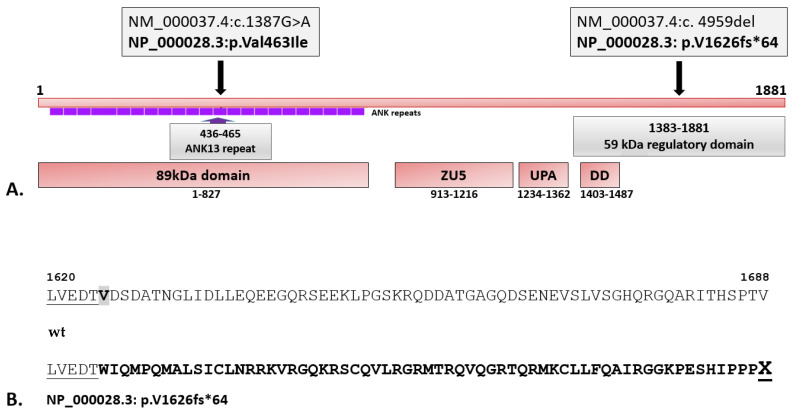
**The location of the two variants detected in the AM family are crucial for the probands.** (**A**) Domain structure of ankyrin-1. The known **missense mutation (NP_000028.3:p.V463I)** was found in the sequence encoding the 89kDa domain (binding site for the anion exchanger AE1), in the region of the ankyrin repeats. The ankyrin ZZUD domain, important for ankyrin binding to spectrin, contains subdomains: ZU5, UPA, and Death domain (DD). The new variant (p.V1626fs*64) is located in the erythrocyte ankyrin regulatory domain sequence. (**B**) A comparison of the amino acid sequence of the wild-type (reference) ankyrin-1 with this containing the detected frameshift mutation, a new single nucleotide deletion (NM_000037.4:c.4959del), resulting in a frameshift and a premature stop codon 64 codons downstream of this change (NP_000028.3:p.V1626fs*64).

In the studied family members (AM174 and AM175) initially diagnosed with HS, the analysis of clinical symptoms and hematological data are in accordance with the WES results. In addition, the typical autosomal dominant type of inheritance of ankyrin-1 defects was observed in this family (OMIM #182900). Mutations in the *ANK1* gene also cause a recessive form of the disease [39], usually with a mild to severe clinical course [1,40]. Ankyrin-1 binds spectrin to AE1 and plays a key role in stabilizing the erythrocyte membrane: therefore, the ankyrin deficiency observed in HS patients leads to a reduction in spectrin assembly at the membrane [37]. Usually, a combined decrease of spectrin and ankyrin is detected (15–50% [1]), which is much easier to prove in HS patients after splenectomy. The ankyrin-1 deficiency in the erythrocyte membranes (that is usually detected by the SDS-PAGE method) can be masked in non-splenectomized patients by excessive hemolysis of the defective erythrocytes and subsequent reticulocytosis [7,41]. As shown by Miraglia et al., normal or even higher-than-normal levels of ankyrin-1 have been observed in patients with HS, which, according to the authors, indicates the inactivation of one ankyrin allele [41]. Two variants in the *ANK1* gene were confirmed to be heterozygous only in HS patients and absent in an asymptomatic AM family member. The detected variants in the *ANK1* gene, a new single nucleotide deletion resulting in a frameshift and a premature stop codon 64 codons downstream (p.V1626fs*64) and a known missense mutation (p.V463I, rs140085544) are most likely to occur in *cis* in both probands, according to the inheritance pattern in the investigated AM family (Figure 3).

Variant p.V463I was found by Eber et al. in a patient with recessive HS, where the second change was detected in the promoter of the *ANK1* gene [31]. The single nucleotide substitution (NM_000037.4:c.-108T>C, rs77173848) is silent in heterozygotes and always co-occurs with another variant of the *ANK1* gene in other HS patients. Interestingly, the missense mutation p.V463I detected by Eber et al. was associated with a decrease in AE1 protein level [31]. This was linked to the variant’s location in the domain of ankyrin, which is responsible for binding the AE1 protein. This most likely involves disrupting the AE1-erythrocyte ankyrin-spectrin complex in the erythrocyte membrane. 

In the AM family, the changes in the transcript are due to the coexistence of the two detected variants (frameshift mutation p.V1626fs*64 and missense mutation p.V463I) that occur in the *cis*. Loss of the double-mutated allele in the cDNA was observed only in the studied patients with HS as was shown for both detected variants (see Figure 2 and Figure S1.1). This indicates that the mutant transcript was not present at a level that could be detected. Therefore, the physiological effects are a consequence of reduced ankyrin-1 expression, leading to membrane dysfunction and premature removal of erythrocytes in the spleen. The clinical manifestation is the hemolytic anemia observed in probands from the AM family. Previously, we have described a similar physiological effect for another frameshift mutation in the *SPTB* gene in HS patients in the Polish population [33]. In other known cases of HS patients with mutations in the *SPTB*, *ANK1*, and *SLC4A1* genes with dominant inheritance, comparable observations have been made [42,43,44,45,46]. 

## 5. Conclusions

Identification of genetic variants that cause rare genetic diseases using high-throughput genomic analysis is now a highly accurate diagnostic tool. It allowed us to detect a novel frameshift mutation (p.V1626fs*64) and a known missense mutation (p.V463I) in the *ANK1* gene in individuals diagnosed with HS. However, using the classical Sanger sequencing method, we could confirm this change in the gDNA and cDNA of the probands. This allowed us to suggest that the mutant transcript is unstable and that the cause of HS symptoms is the decrease in ankyrin-1 protein. This diagnostic approach allows us to verify the reliability of genome analysis methods using NGS and to deepen our knowledge of the molecular basis of a rare genetic disorder.

## Data Availability

All data generated or analyzed during this study are included in this article and its additional files. WES datasets used and/or analyzed during this study are available from the corresponding author upon reasonable request.

## Supplementary Materials

The following supporting information can be downloaded at: https://www.mdpi.com/article/10.3390/biomedicines13020308/s1, Additional file S1: Supplementary tables and figure. This file contains supplementary materials: Table S1.1: Sequences of the PCR primers designed to verify the variants detected in the *ANK1* gene (NC_000007.14). Table S1.2: The PCR primer sequences used to amplify the *ANK1* gene transcript variant 3 (NM_000037.4, MANE). Table S1.3: Variants identified by Sanger sequencing of all genes studied in AM family members. *Accessed on 27 November 2024 Table S1.4: Components of the PCR Reaction Mixture. Table S1.5: PCR (touch-down) reaction conditions. Table S1.6: Hematological parameters of probands from the AM family: white blood cells (WBCs); red blood cells (RBCs); hemoglobin (Hb); hematocrit (HCT); platelets (PLTs); mean corpuscular volume (MCV); mean corpuscular hemoglobin (MCH); mean corpuscular hemoglobin concentration (MCHC). Figure S1.1: Fragment of sequencing traces showing localization of the missense mutation (p.V463I, rs140085544). The mutation is not present in a healthy individual (AM173). A heterozygous single nucleotide substitution G/A is identified in the HS patient AM174 (genomic DNA was used as a template). Loss of the mutant allele is shown in the cDNA relative to the genomic DNA (cDNA from patient AM174 was used as a template). The sequence compatible with the reference sequence is indicated by the green color of the boxes, the red color indicates the position of the pathogenic variant in the sequence. Additional file S2: Supplementary tables. Detailed data on the analyzed variants detected using WES (in the panel of genes recommended for RBC pathologies and analyzed using the Ingenuity Variant Analysis plugin (IVA; QIAGEN, Redwood City, CA, USA). Additional file S3: Detailed data on the analyzed variants detected by WES in the genes whose mutations result in HS and analyzed using the Ingenuity Variant Analysis plugin (IVA; QIAGEN, Redwood City, CA, USA).
