# Supplementary material for: Two Variants of the ANK1 Gene Associated with Hereditary Spherocytosis
Source: Biomedicines. 2025 Jan 27;13(2):308. doi: 10.3390/biomedicines13020308 (PMC11853173; doi:10.3390/biomedicines13020308)
Supplement: Supplementary file 1 [file biomedicines-13-00308-s001.zip › biomedicines-3431635_Additional file 1-xml.pdf]

<u>**Additional file 1**</u>

Supplementary Materials for Article:

Two Variants of the *ANK1* Gene Associated with Hereditary Spherocytosis.

Bogusławska et. al

Table S1.1 Sequences of the PCR primers designed to verify the variants detected in the *ANK1* gene (NC_000007.14).

PCR primers	Sequence (5'->3')	Product size	Description of variant
ANK1gDNAc.4959delS	TAGCAAGGCTGAGGACTCTGATGC	482	**new variant**<br>NC_000008.11:g.41672575del<br>NM_000037.4(ANK1):c. 4959del<br>NP_000028.3: p.V1626fs*64
ANK1gDNAc.4959delA	CTGCCAGACTGAATGTATGTGCTCC		
ANK1gDNAc.1387G>AS	TGCACACATACCAGGCTAAAGAGC	666	**rs140085544**<br>NC_000008.11:g.41716970C>T<br>NM_000037.4(ANK1):c.1387G>A<br>NP_000028.3: p.Val463Ile
ANK1gDNAc.1387G>AA	CTTCTCACTGTCTACTCCTCCAACC		

Table S1.2 The PCR primer sequences used to amplify the *ANK1* gene transcript variant 3 (NM_000037.4, MANE).

PCR primers	Sequence (5'->3')	Product size	Description of variant
ANK1cDNAc.4959delS	TCTCTGGTCACTGCTGAGGACTCC	517	**new variant**<br>NC_000008.11:g.41672575del<br>NM_000037.4(ANK1):c. 4959del<br>NP_000028.3: p.V1626fs*64
ANK1cDNAc.4959delA	CTGCCAGACTGAATGTATGTGCTCC		
ANK1cDNAc.1387G>AS	ACCCAACTCCAGAGCCCTGAATGG	678	**rs140085544**<br>NC_000008.11:g.41716970C>T<br>NM_000037.4(ANK1):c.1387G>A<br>NP_000028.3: p.Val463Ile
ANK1cDNAc.1387G>AA	AGCAGCAGCTTGACGATGTCCAGG		

Table S1.3 Variants identified by Sanger sequencing of all genes studied in AM family members (hom – homozygotic het – heterozygotic; abs – absence). *accessed on 27 November 2024

Gene name	Reference SNP ID number/ HGMD ID	Change of nucleotide/ amino acid residue	Inheritance / probands (WES) Family member AM173	HS patients AM174	HS patients AM175	ClinVar* Clinical Significance/ Variation ID
			**Membranopaties**			
ANK1	new variant	NC_000008.11:g.41672575del<br>NM_000037.4:c. 4959del<br>**NP_000028.3: p.V1626fs*64**	abs	het	het	Not Reported in ClinVar
ANK1	rs140085544/ CM960064	NC_000008.10:g.41574488C>T<br>NM_000037.4:c.1387G>A<br>**NP_000028.3:p.Val463Ile**	abs	het	het	Conflicting classifications of pathogenicity<br>Uncertain significance(2); Benign(1); Likely benign(1)<br>ID: 719959
SPTB	rs2082307149	NC_000014.8:g.65240045G>A<br>NM_001355436.2:c.5071C>T<br>**NP_001342365.1:p.His1691Tyr**	abs	abs	het	Not Reported in ClinVar
SPTB	rs17180350/ CM187439 (DM?)	NC_000014.8:g.65249066C>T<br>NM_001355436.2:c.4208G>A<br>**NP_001342365.1:p.Arg1403Gln**	hom	het	abs	Benign/Likely benign<br>ID: 257114
TRPV1	rs222747	NC_000017.10:g.3493200C>G<br>NM_080704.4:c.945G>T<br>**NP_061197.4:p.Met315Ile**	abs	het	het	Not Reported in ClinVar
			**Enzymopaties**			
ZFPM1	rs1491526237	NC_000016.9:g.88599697_88599698del<br>NM_153813.3:c.1331_1332del<br>**NP_722520.2:p.Leu446_Ala447delinsPro**	hom	hom	hom	Not Reported in ClinVar
	rs67322929	NC_000016.9:g.88599701del<br>NM_153813.3:c.1335del<br>**NP_722520.2:p.Leu446fs**	hom	hom	hom	Not Reported in ClinVar
	rs67873604	NC_000016.9:g.88599703_88599705del<br>**NM_153813.3:c.1337_1339del**<br>**NP_722520.2:p.Glu444fs**	hom	hom	hom	Not Reported in ClinVar
			**Other potentially pathogenic variants**			
BRCA1	rs80357906	NC_000017.10:g.41209082dup<br>NM_007294.4:c.5266dup<br>**NP_009225.1:p.Gln1756fs**	het	abs	abs	Pathogenic<br>ID: 17677
ENAM	rs587776588	NC_000004.11:g.71508402_71508403insAG<br>NM_031889.3(ENAM):c.1259_1260insAG<br>**NP_114095.2:p.Pro422fs*27**	abs	het	het	Pathogenic/Likely pathogenic<br>ID: 4238
FGA	new variant	NC_000004.12.: 155507590-155507591delTA	het	abs	abs	Not Reported in ClinVar
FGA	rs6050	NC_000004.12:g.154586438T>C<br>NM_021871.4(FGA):c.991A>G<br>**NP_068657.1:p.Thr331Ala**	hom	hom	het	Conflicting classifications of pathogenicity<br>Uncertain significance(1); Benign(4)<br>ID: 16420

Table S1.4 Components of the PCR Reaction Mixture.

Component	Final concentration
**HOT FIREPol® DNA Polymerase (01-02-KIT-0000S) Solis BioDyne OÜ, Tartu, Estonia (EU)**	**0.05 U/µl**
HOT FIREPol® 10x Buffer B1	1x
25 mM MgCl2	5 mM
10 mM dNTP MIX	200 µM of each
Forward Primer (10 µM)	10 nM
Reverse Primer (10 µM)	10 nM
10x GC-rich Enhancer (optional)	1x
Template DNA (gNDA/cDNA)	0.1-1 µg/50 µl
Nuclease-free water	up to 50 µl
**Total reaction volume**	**50 µl**

Table S1.5 PCR (touch-down) reaction conditions.

No.	Steps	Temperature	Time [s]	Cycles
1.	Initial activation	95°C	900	
2.	Denaturation	95°C	35	
3.	Annealing	64/67°C	40	
4.	Extension	72°C	40-60	3
5.	Denaturation	95°C	35	
6.	Annealing	63/66°C	40	
7.	Extension	72°C	40-60	5
8.	Denaturation	95°C	35	
9.	Annealing	62/65°C	40	
10.	Extension	72°C	40-60	6
11.	Denaturation	95°C	35	
12.	Annealing	61/64°C	40	
13.	Extension	72°C	40-60	30-33
14.	Final extension	72°C	600	

Table S1.6. Hematological parameters of probands from the AM family: white blood cells (WBCs); red blood cells (RBCs); hemoglobin (Hb); hematocrit (HCT); platelets (PLTs); mean corpuscular volume (MCV); mean corpuscular hemoglobin (MCH); mean corpuscular hemoglobin concentration (MCHC).

Laboratory tests	Units	HS patients		Reference range (female)	Reference range (male)
		AM174 (female)	AM175 (male)		
WBC	(G/L)	11.37±3.02	11.80±2.05	4–10	4–10
RBC	(T/L)	3.58±0.35	3.58±0.35	4.0–5.0	4.5–5.9
Hb	(mmol/L)	7.21±0.50	7.65±0.52	7.54–9.93	8.69–11.17
HCT	(L/L)	0.32±0.02	0.34±0.02	0.37–0.47	0.37–0.53
PLT	(G/L)	321±20	222±19	140–440	140–440
MCV	(fL)	88±2	95±3	81–98	81–98
MCH	(fmol)	2.02±0.06	2.17±0.13	1.61–2.11	1.61–2.11
MCHC	(mmol/L)	22.88±0.11	21.16±2.14	19.24–22.96	19.24–22.96

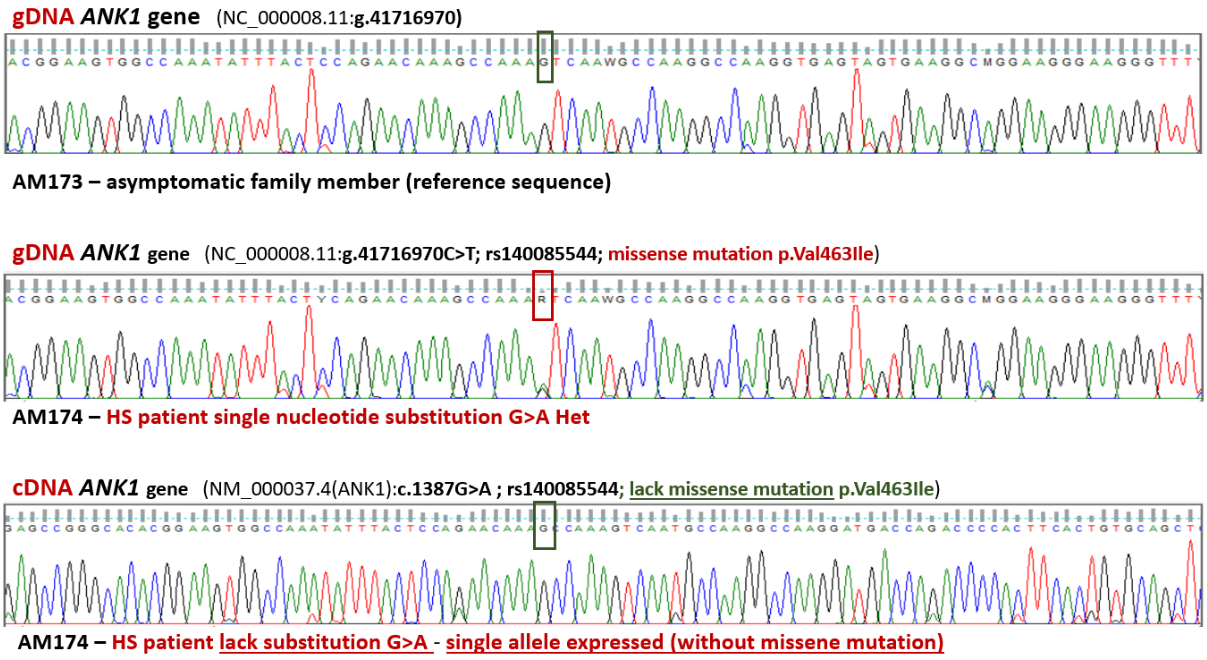

Figure S1.1 Fragment of sequencing traces showing localization of the missense mutation (p.V463I, rs140085544). The mutation is not present in a healthy individual (AM173). A heterozygous single nucleotide substitution G/A is identified in the HS patient AM174 (genomic DNA was used as a template). Loss of the mutant allele is shown in the cDNA relative to the genomic DNA (cDNA from patient AM174 was used as a template). The sequence compatible with the reference sequence is indicated by the green color of the boxes, the red color indicates the position of the pathogenic variant in the sequence.
